# Supplementary material for: Identification and functional analysis of non-coding regulatory small RNA FenSr3 in Bacillus amyloliquefaciens LPB-18
Source: PeerJ. 2023 May 15;11:e15236. doi: 10.7717/peerj.15236 (PMC10194069; doi:10.7717/peerj.15236)
Supplement: Supplemental Information 4 [file peerj-11-15236-s004.zip › KO/CK-vs-T1_map/map04111.html]

KEGG PATHWAY: Cell cycle - yeast - Reference pathway


|  |  |
| --- | --- |
| **Cell cycle - yeast - Reference pathway** |  |

[
Pathway menu
| Organism menu
| Pathway entry
| Show description
| User data mapping
]

Mitotic cell cycle progression is accomplished through a reproducible sequence of events, DNA replication (S phase) and mitosis (M phase) separated temporally by gaps (G1 and G2 phases). G1, S, and G2 phases are collectively known as interphase. In yeast Cdc28 is the catalytic subunit of the cyclin-dependent kinase (CDK). At G1 phase Cdc28 associates with G1-cyclins Cln1 to Cln3, while B-type cyclins Clb1 to Clb6 regulate Cdc28 during S, G2, and M phases. Cln3/Cdc28 activity is required for cells to pass through 'start', the commitment point in G1. When Cln3/Cdc28 accumulates more than a certain threshold, SBF (Swi4/Swi6) and MBF (Mbp1/Swi6) are activated, promoting transcription of Cln1, Cln2, and other genes required for S-phase progression. Cln1 and Cln2 interacting with Cdc28 promote activation of B-type cyclin associated CDK, which drives DNA replication and entry into mitosis. Specifically, Cdc28 association with Clb2 and Clb1 promotes entry into mitosis. Cells suffering from DNA damage, spindle misorientation, or spindle assembly defect do not undergo the metaphase-anaphase transition for chromosome segregation and fail to exit from mitosis. The spindle assembly checkpoint activates Mad2, which in turn prevents chromosome segregation by inhibiting degradation of the securin Pds1. Moreover, Pds1 is phosphorylated and stabilized in response to DNA damage in a Chk1-dependent manner. The spindle checkpoint is also involved in the Cdc14 release from the nucleolus. Cdc14 dephosphorylates Swi5, Sic1, and Cdh1, leading to inhibition of Cdc28 and degradation of cyclin required for mitotic exit.

|  |  |  |
| --- | --- | --- |
| Reference pathway | 184% 150% 122% 100% 82% 67% 55% | 图片下载 |
